# Supplementary material for: Luminescent Studies on Germanate Glasses Doped with Europium Ions for Photonic Applications
Source: Materials (Basel). 2020 Jun 23;13(12):2817. doi: 10.3390/ma13122817 (PMC7345945; doi:10.3390/ma13122817)
Supplement: Supplementary file 1 [file materials-13-02817-s001.pdf]

materials

MDPI

Supplementary Materials

Luminescent Studies on Germanate Glasses Doped with Europium Ions for Photonic Applications

Jacek Żmojda[1,*], Marcin Kochanowicz [1], Piotr Miluski [1], Piotr Golonko [1], Agata Baranowska [2], Tomasz Ragiń [2], Jan Dorosz [1], Marta Kuwik [3], Wojciech Pisarski [3], Joanna Pisarska [3], Renata Szal [4], Gabriela Mach [4], Bartosz Starzyk [4], Magdalena Leśniak [4], Maciej Sitarz [4] and Dominik Dorosz [4]

[1] Faculty of Electrical Engineering, Bialystok University of Technology, 45D Wiejska Street, 15-351 Bialystok, Poland; m.kochanowicz@pb.edu.pl (M.K.); p.miluski@pb.edu.pl (P.M.); piotr@neoweb.pl (P.G.); doroszjan@pb.edu.pl (J.D.)

[2] Faculty of Mechanical Engineering, Bialystok University of Technology, 45C Wiejska Street, 15-351 Bialystok, Poland; a.baranowska@pb.edu.pl (A.B.); t.ragin@pb.edu.pl (T.R.)

[3] Institute of Chemistry, University of Silesia, 9 Szkolna Street, 40-007 Katowice, Poland; marta.kuwik88@gmail.com (M.K.); wojciech.pisarski@us.edu.pl (W.P.); joanna.pisarska@us.edu.pl (J.P.)

[4] Faculty of Materials Science and Ceramics, AGH University of Science and Technology, 30 Mickiewicza Av., 30-059 Krakow, Poland; renata.szal@agh.edu.pl (R.S.); machgabriela1@gmail.com (G.M.); bar.s@wp.pl (B.S.); mlesniak@agh.edu.pl (M.L.); msitarz@agh.edu.pl (M.S.); ddorosz@agh.edu.pl (D.D.)

* Correspondence: j.zmojda@pb.edu.pl; Tel.: +48-692-995-305

Received: 15 May 2020; Accepted: 16 June 2020; Published: 23 June 2020

Powder X-ray Diffraction Analysis

Powder X-ray diffraction analysis of samples was performed using X'Pert Pro X-ray diffractometer supplied by PANalytical equipped with a copper anode CuKα1 radiation (λ = 1.54056 Å, 40 kV, 40 mA) positioned in Bragg-Brentano geometry.

The beam focusing by the Bragg-Brentano method consisted of simultaneously placing the radiation source, sample, and detector on one focus circle with a variable radius r. In the case of the Bragg-Brentano method X-rays are incident on the sample at an angle θ and the counter must be in a position 2θ angle to the direction of the incident beam. To increase the number of different positions of lattice planes in crystallites, each sample was rotated during the measurement. The rotation changes of the θ angle value, so to maintain the θ–2θ relationship throughout the measurement for sample and counter, respectively, the counter moved around the circumference of the goniometer with the speed two times faster than the sample was rotated. The counter position was specified as a function of the 2θ angle, at the same time determining the position of the reflections on the diffractogram as a function of the same angle (Figure S1).

The measured 2θ angle range was 5°–90° with a step size of 0.017°, time per step of 184.79 s, and a scan speed of 0.011 °/s. The samples were prepared by the backloading method. The measured data were treated for interpretation using the HighScore program.

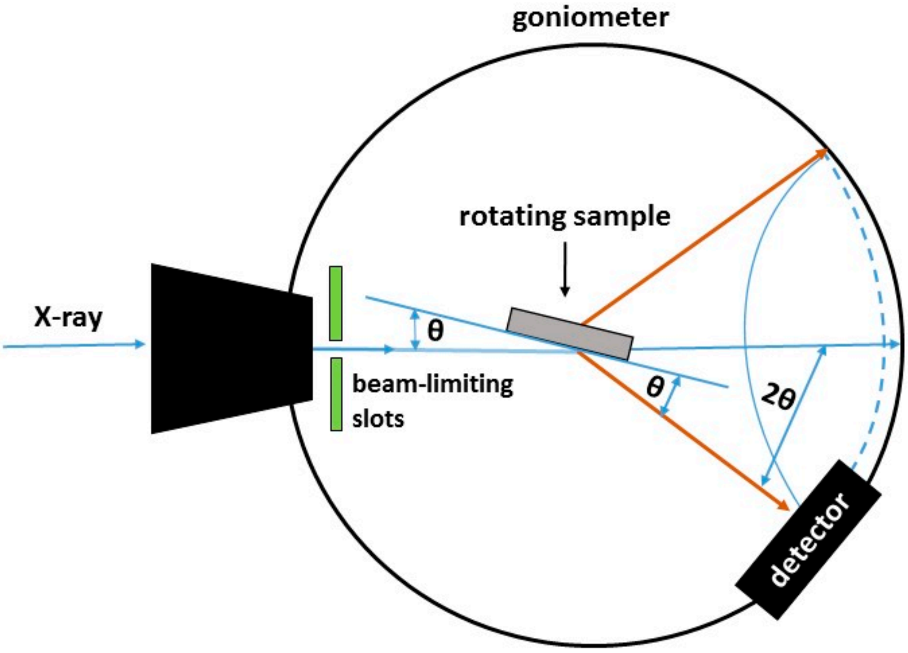

Figure S1. Schematic diagram of the XRD set-up.

© 2020 by the authors. Licensee MDPI, Basel, Switzerland. This article is an open access article distributed under the terms and conditions of the Creative Commons Attribution (CC BY) license (http://creativecommons.org/licenses/by/4.0/).
